# Pediatric nasal dermoid sinus cysts: advances in pathogenesis, management strategies, and translational research—a multidisciplinary management perspective

**DOI:** 10.3389/fped.2025.1708853

**Published:** 2026-01-09

**Authors:** Shan Quan, Li He, Jiangqiao Geng, Juan Wang

**Affiliations:** Ear, Nose and Throat Department, Children’s Hospital of Hebei Hospital, Hebei Clinical Medicine Research Center for Children’s Health and Diseases, Shijiazhuang, Hebei, China

**Keywords:** nasal dermoid/sinus cysts, pathogenesis, diagnostic and therapeutic strategies, multidisciplinary management, clinical translation

## Abstract

**Background:**

Pediatric nasal dermoid/sinus cysts (NDSC) are common congenital midline craniofacial lesions associated with embryonic tissue entrapment and abnormal midline fusion during embryogenesis; they can involve intracranial structures and predispose patients to recurrent infection, intracranial complications, and cosmetic or functional impairment, posing challenges for diagnosis, treatment, and long-term management.

**Objective:**

To review the pathogenesis, diagnostic and therapeutic strategies, and advances in clinical translation of NDSC, and to explore optimization of care under multidisciplinary management.

**Methods:**

We systematically reviewed recent literature on embryology, imaging, surgical techniques, and multidisciplinary team (MDT) approaches.

**Results:**

Aberrant midline fusion and ectodermal inclusion during embryogenesis represent the principal pathogenic theories; lesions may be classified as superficial, sinus-tract, or those with intracranial extension. MRI is the imaging modality of choice, CT is used for osseous assessment, and ultrasound and endoscopy serve as bedside and intraoperative adjuncts. Treatment centers on complete excision: external rhinoplasty approaches, endoscopic endonasal techniques, or combined intracranial–extracranial approaches should be individualized according to imaging-based classification; perioperative infection control and standardized follow-up substantially reduce recurrence. In clinical translation, intraoperative navigation, radiomics, and MDT collaboration have improved operative safety and cosmetic outcomes, whereas studies addressing molecular mechanisms, biomarkers, and long-term functional prognosis remain insufficient.

**Conclusion:**

We recommend implementing individualized surgical strategies guided by imaging classification and advancing MDT decision pathways; future work should intensify molecular and radiomics research to achieve more precise diagnosis, therapeutic selection, and long-term prognostic assessment.

## 1 Introduction

Pediatric nasal dermoid/sinus cysts (NDSC) represent a group of rare congenital midline nasal lesions that typically present in early childhood and commonly manifest as a painless mass, sinus tract, or fistulous opening at the nasal root or dorsum (1, 2). The overall incidence is low, which contributes to limited clinical awareness, and the abnormality carries important implications for pediatric otolaryngology and head and neck surgery (2). Because clinical signs and external appearance are often non-specific, these lesions are frequently misdiagnosed as other common nasal conditions, leading in some cases to delayed diagnosis and treatment and thereby increasing the risk of infection, surgical morbidity, and recurrence (2, 3). It is important to note that although the majority of cases are confined to the nasal region, a subset of children may exhibit intracranial extension; approximately 10% of midline nasal dermoid cysts carry this risk, with involvement ranging from epidural to intradural compartments (4, 5). Such variants can necessitate more complex surgical management and may result in severe complications, including meningitis, intracranial abscess, or other intracranial infections (4).

At present, an embryologic fusion-defect hypothesis is widely cited to explain the pathogenesis of nasal dermoid/sinus cysts, yet the underlying molecular and developmental pathways remain incompletely defined and current theories have limitations (6). From a diagnostic standpoint, imaging—particularly magnetic resonance imaging (MRI) and computed tomography (CT)—plays a central role in assessing lesion extent and screening for intracranial extension. However, discrepancies frequently exist between preoperative imaging and intraoperative findings; imaging modalities have limited accuracy in very young children, and standardized imaging criteria are lacking, especially reducing diagnostic value in infants and toddlers (4, 7). Indications for combined intracranial approaches likewise remain unsettled: some studies advocate individualized strategies based on the scope of lesion extension and the degree of bony involvement, but concrete operative thresholds and consensus criteria are still debated (1, 5).

Moreover, selecting a surgical approach that appropriately balances cosmetic outcomes, recurrence risk, and complication rates remains a central challenge for multidisciplinary teams. Recent systematic reviews suggest that external incision approaches are associated with the lowest recurrence and complication rates, whereas the long-term cosmetic and functional outcomes of rhinoplasty-based and endoscopic techniques are not yet well established; therefore, choice of operative route should integrate lesion characteristics, patient age, and cosmetic considerations (8). To overcome current diagnostic and therapeutic dilemmas, a systematic synthesis of the epidemiology, pathogenesis, management strategies, and translational research progress—with emphasis on multidisciplinary collaboration and optimization of treatment decisions—is warranted. This review aims to provide the latest evidence and practical guidance to support clinical recognition, individualized management, and multidisciplinary care of pediatric nasal dermoid/sinus cysts.

## 2 Methodology

This review was conducted in strict accordance with the PRISMA (Preferred Reporting Items for Systematic Reviews and Meta-Analyses) guidelines. Literature searches were performed in PubMed, Embase, and the Cochrane Library, with the retrieval period defined as January 2023 to August 2025, and the publication window for included studies limited to 2013–2025. The search strategy combined keywords such as “nasal dermoid sinus cyst”, “embryology”, and “multidisciplinary management”, constructed using Boolean operators (AND, OR, NOT), and was restricted to publications in English and Chinese. To enhance specificity, non-original research items—such as conference abstracts, review articles, and studies whose full texts were not obtainable—were excluded.

Study selection was carried out independently by two reviewers according to predefined criteria and proceeded through three stages: title screening, abstract screening, and full-text review. Inclusion criteria were: ① studies with a clear diagnosis of nasal dermoid sinus cyst; ② studies addressing pathogenesis, diagnostic or therapeutic strategies, or translational clinical advances; ③ studies discussing aspects related to multidisciplinary management. Exclusion criteria included: ① publications not in English or Chinese; ② duplicate reports or studies with incomplete data; ③ articles unrelated to the study topic. Any disagreements between the two reviewers during screening were resolved by arbitration from a third expert to achieve consensus. To ensure the quality of included studies, a modified Newcastle–Ottawa Scale (NOS) was used to assess case series and retrospective studies. The assessment encompassed multiple dimensions, including the representativeness of case selection, the scientific rigor of study design, and the reliability of outcome assessment. Data extraction was performed using a standardized form and captured key information such as study design, number of cases, diagnostic methods, therapeutic strategies, and follow-up results. Finally, based on the data extracted from the included studies, a qualitative synthesis was undertaken to systematically summarize the pathogenesis, diagnostic and therapeutic strategies, and translational clinical advances related to nasal dermoid sinus cysts; these findings were integrated with existing theoretical frameworks and clinical practice to provide recommendations for optimizing care under a multidisciplinary management paradigm.

## 3 Pathogenesis

The pathogenesis of nasal dermoid/sinus cysts is closely linked to their embryologic basis; fundamentally, these lesions arise from abnormal incorporation of ectodermal elements into the midline during early embryogenesis due to defective fusion of midline structures (3). During the coordinated development of frontonasal structures such as the frontonasal prominence and the nasal plates, dysregulated fusion of the frontonasal prominence or related prominences can readily result in persistence of ectodermal cells at the midline, thereby creating a substrate for subsequent dermoid cyst or sinus formation (3, 6). Mutations in, or dysregulated expression of, key molecules such as ALX1 and SIX2 may also produce mesenchymal developmental disturbances in the frontonasal region, triggering midline craniofacial abnormalities and fissures; however, no direct evidence currently establishes a specific association between these genetic abnormalities and nasal dermoid/sinus cysts, indicating that the molecular mechanisms require further elucidation (9, 10). The principal pathogenic theories at present include the ectodermal inclusion theory and the embryonic closure-defect theory (3, 6). The ectodermal inclusion theory explains how, during fusion of facial prominences, small portions of surface ectoderm may become entrapped and later differentiate into dermoid tissue, accounting for the characteristic midline superficial cysts and sinuses commonly seen in children (3). By contrast, the embryonic closure-defect theory emphasizes a more extensive failure of midline seam closure during frontonasal development, a mechanism that can predispose to both localized cyst formation and the development of deeper epithelialized tracts that in some cases track posteriorly toward the anterior cranial fossa (6). Together, these theories explain the spectrum from superficial dermoid cysts to lesions with potential intracranial extension.

Histologically, nasal dermoid/sinus cysts typically exhibit a cyst wall lined by keratinizing squamous epithelium and may contain adnexal structures such as sebaceous glands and hair follicles, reflecting their origin from ectopic ectodermal components during embryogenesis (3, 11). In some cases, thyroid tissue or respiratory epithelium can be admixed within the cyst wall, further illustrating the developmental mingling of multiple midline tissues during embryogenesis and partially supporting theories of ectodermal inclusion and multi-tissue fusion abnormalities (12). The sinus-type lesions tend to reflect incomplete closure of a fistulous tract during embryonic development, whereas the cyst-type lesions represent localized encapsulation and aberrant differentiation of ectodermal tissue (3, 11). A subset of cases demonstrates deep extension, traversing the nasal bones toward the intracranial compartment, suggesting that defects in cartilage formation and ossification-center closure during embryogenesis may permit persistence of deep adnexal structures and the formation of complex anterior cranial base tracts or cysts (5, 6). When intracranial extension is present, it often involves a persistent communication at the dura–osseous interface; these cases present with complex clinical manifestations, greater surgical difficulty, and higher risk of recurrence and secondary infection (2, 5). Moreover, types associated with intracranial extension frequently indicate more severe midline closure defects during development and must be differentiated from encephalocele and other midline structural malformations (6, 11). Embryological and molecular investigations have yielded important clues regarding complex midline nasal anomalies, but with respect to the core pathogenesis of nasal dermoid/sinus cysts, abnormal tissue fusion and ectodermal entrapment remain the dominant paradigms; direct molecular-level evidence is still lacking, and most current understanding relies on anatomical, histological, and clinicopathological data.

## 4 Clinical presentation and classification

**Figure 1:**
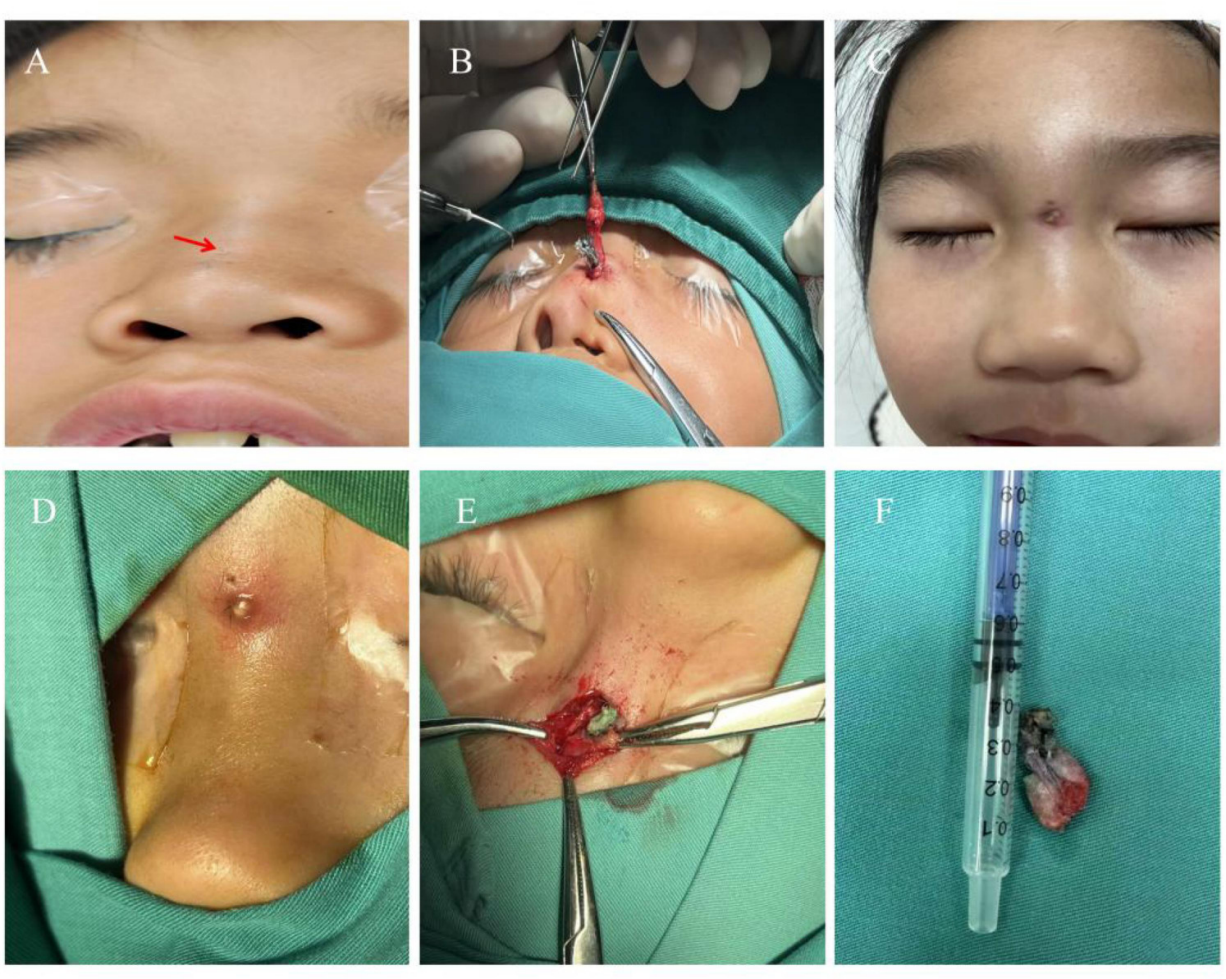
Common clinical manifestations of children with nasal dermoid sinus cysts. **(A)** Preoperative appearance, a fistula can be seen on the dorsal side of the nose (indicated by an arrow); **(B)** Intraoperative field of view. After the fistula was opened, white keratinized secretions and hair could be seen. **(C)** Preoperative appearance, repeated local infections on the dorsal side of the nose, accompanied by skin redness and swelling. **(D)** Intraoperative local view, showing slight congestion and swelling on the dorsal part of the nose, with a few hairs. **(E)** Intraoperative field of view, visible white secretions and a few hairs; **(F)** A complete fistula was excised during the operation (blue indicates staining with Meilan contrast agent).

The clinical presentation of pediatric nasal dermoid/sinus cysts exhibits certain characteristic features; classically, affected children present with a painless mass or localized protuberance located on the midline of the nasal root or dorsum (13, 14) (see Table 1 and Figure 1). Some patients show a cutaneous sinus opening, and a minority of cases demonstrate exposed hair—an emblematic sign of the condition (14, 15). When an open sinus tract exists, periodic or continuous discharge may be observed; some children develop local dimpling or swelling, and secondary infection can further aggravate local findings, including erythema, pain, or even abscess formation (16). Furthermore, cysts or sinuses may be found at various sites along the nasal dorsum, with variable anatomic locations but predominantly involving the middle-to-upper one-third of the dorsum (15). In rare instances the cyst or tract may traverse bony structures and extend intracranially, producing a more complex presentation and potentially precipitating severe intracranial infection or neurological symptoms (4). Classification of nasal dermoid/sinus cysts is primarily based on anatomical and radiological features; commonly used classification systems divide lesions into superficial, sinus-tract (fistulous), and intracranially-extending types (13, 15). The superficial type is typically confined to the subcutaneous tissue or the fascial layer of the nasal dorsum and presents as an isolated mass without an obvious sinus tract or deep adhesions; the sinus-tract type is characterized by a midline-transversing tract often accompanied by a cutaneous opening and/or exposed hair, and is prone to recurrent infection or persistent drainage (14, 17). The intracranially-extending type denotes lesions that pass through cartilaginous defects or bony gaps of the anterior cranial base, with potential involvement of the intracranial epidural or intradural compartments and occasional major complications such as cerebrospinal fluid leak or meningitis (4). Clinical manifestations differ between types: the superficial type most often presents as an asymptomatic, localized mass; the sinus-tract type, owing to its cutaneous opening and discharge, is more susceptible to infection and in some patients to recurrent suppuration; the intracranial-extending type is characterized by recurrent or persistent infection and carries a risk of neurological complications (4, 13). Some reports indicate that infants and toddlers more commonly present with local prominence or sinus tracts, whereas school-aged children and older patients are more likely to be found to have larger cysts or to seek care because of infectious symptoms, suggesting age-related variation in clinical presentation (15). In the differential diagnosis, entities such as nasal glioma, encephalocele, and epidermoid cyst must be distinguished from nasal dermoid/sinus cysts—particularly because intracranial-related manifestations and imaging features may overlap, posing diagnostic challenges (3, 15). Classification not only facilitates accurate clinical identification and risk assessment but also provides an important foundation for devising individualized surgical strategies and prognostic judgments, especially when deciding whether combined intracranial intervention is required (4, 15).

**Table 1:** Pathogenesis, clinical phenotypes and imaging features of pediatric nasal dermoid/sinus cysts.

Type	Mechanism (embryology/pathological highlights)	Typical clinical presentation	Typical age at detection/presentation	Corresponding imaging features (MRI/CT/US)	Pathology/Differential diagnosis	Notes (preferred imaging/clinical recommendations)
Superficial type (superficial)	Ectodermal epithelial elements become trapped in the subcutaneous tissues during embryogenesis, forming an epithelial-lined cyst that accumulates keratinaceous debris and sebum; secondary infection or rupture may exacerbate symptoms.	Palpable, mobile subcutaneous mass that is painless or minimally tender on pressure; no obvious external sinus opening; occasional local erythema, discharge or recurrent infection.	Birth—3 years (most detected at 0–2 years)	MRI: cyst contents containing fat are T1 hyperintense and suppress on fat-suppressed sequences; T2 signal typically high; cyst wall or adjacent inflammation may enhance on postcontrast images. CT: soft-tissue density or low CT values if fat is present; bone usually shows compressive rather than destructive changes. US: well-defined cystic anechoic/hypoechoic structure; internal scattered echoes may reflect fat or debris; useful for point-of-care differentiation.	Epidermoid cyst, pilonidal/follicular cysts, other follicular lesions	Superficial lesions—initially evaluate with ultrasound for bedside assessment; if deep extension is suspected or for preoperative planning, perform MRI with fat-suppressed and contrast sequences.
Sinus/Tract type (Sinus/Tract)	During embryonic fusion, ectodermal tissue is carried inward and maintains a communication with the skin surface, creating a skin-to-deep tract; keratin and secretions within the tract predispose to bacterial contamination and recurrent infection/tract expansion.	Small skin pit or pore on the surface, intermittent sticky or purulent discharge and recurrent infections/tract inflammation.	Birth—5 years (most detected at 0–3 years)	MRI: demonstrates a tract extending from the skin to deeper tissues; fat-saturated sequences help distinguish fatty components; contrast enhancement can show the tract wall and peritract inflammation. CT (bone window): may show focal bony defects or changes along the tract. Endoscopy: can directly visualize the external opening but cannot evaluate intracranial course.	Congenital nasal cysts (e.g., naso-frontal cyst), congenital follicular sinuses	When a sinus/tract or recurrent infection is suspected, MRI to map the entire tract is preferred; thin-slice CT should be added to assess bony defects when surgical planning requires it.
Intracranial extension type (Intracranial extension)	Developmental anomalies of the nasal root/cribriform plate/anterior skull base or bony defects permit ectodermal remnants to extend through bone defects or soft-tissue channels into the cranial cavity; intracranial extension may be promoted by cyst pressure or infection.	May present only as a nasal-root/nasal-dorsum prominence without an external pit; if infected, systemic signs (fever) or signs of intracranial infection/meningeal irritation may occur (rare).	Birth—12 years (most cases detected/suspected at 0–2 years)	MRI (preferred): demonstrates lesion extension through cribriform plate/nasal bone defects into the anterior cranial fossa; may appear as extradural, subdural or intradural/hard-meningeal masses. Fat components are T1 hyperintense and suppress on fat-suppressed sequences; contrast enhancement may delineate cyst wall/tract and relationship to dura/brain surface. CT (thin-slice bone window): used to identify and quantify skull-base/nasal bone defects and for 3-D reconstruction to aid surgical planning. Ultrasound: insufficient to exclude intracranial extension.	Nasal glioma, encephalocele, other anterior cranial fossa congenital lesions or neoplasms (requires imaging differentiation)	In cases with high suspicion of intracranial extension, prioritize MRI with contrast and fat-suppressed sequences plus thin-slice bone-window CT for comprehensive preoperative assessment and surgical planning. Early/confirmed intracranial cases should be managed by a multidisciplinary team (ENT, neurosurgery, plastics).

MRI, magnetic resonance imaging; CT, computed tomography; US, ultrasound; MDT, multidisciplinary team; ENT, ear, nose & throat (otorhinolaryngology).

## 5 Imaging diagnosis

The aim of preoperative imaging assessment for pediatric nasal dermoid/sinus cysts is to delineate the lesion's extent and type and to define its relationships with adjacent anatomical structures, thereby providing an evidence base for surgical planning (4). A systematic, multimodal preoperative imaging work-up not only enhances diagnostic accuracy but also enables optimization of individualized surgical strategies and reduction of procedure-related risks. Table 2 summarizes the indications and limitations of each imaging modality in pediatric nasal dermoid/sinus cysts.

**Table 2:** Clinical uses and limitations of imaging modalities for pediatric nasal dermoid/sinus cysts.

Imaging/examination modality	Primary purpose (clinical question)	Key sequences/windows/technical parameters	Limitations	Indications (recommendation)
MRI	Evaluate soft-tissue components (fat/keratin), map sinus/tract course, assess relationship to dura/brain surface, and preoperative evaluation for intracranial extension	T1 (with and without fat suppression), T2WI, fat-suppressed T1 or STIR, post-contrast T1, 3D high-resolution sequences (thin-slice axial/sagittal/coronal)	Relatively insensitive to very small bony defects (<∼1–2 mm)—complementary CT often needed. Children frequently require sedation/general anesthesia to control motion artifacts (especially <6 years or uncooperative), which carries anesthetic risk and increases time/cost. Signal characteristics may be variable for high-protein or hemorrhagic contents; fat-suppressed sequences are necessary to identify fat and avoid misinterpretation.	First-line for suspected intracranial extension and for detailed preoperative mapping of soft tissue and tract anatomy. Include fat-suppression sequences when fatty cyst contents are suspected.
CT (thin-slice + bone window)	Quantitative assessment of bone, detection of nasal bone/cribriform plate/anterior skull-base defects, and 3-D reconstruction for surgical approach planning	≤1.0 mm thin-slice reconstruction; bone window; 3-D surface/volume reconstructions (ensure consistent patient positioning for intraoperative registration)	Involves ionizing radiation: use pediatric low-dose protocols and carefully balance indications (routine screening CT is not recommended). Poor soft-tissue contrast for fat/inflammatory fluid—often requires correlation with MRI. Reconstruction quality is degraded by motion and metal artifacts.	Preferred when precise delineation or quantification of skull-base/nasal bone defects is required, for intraoperative navigation registration, or for 3-D surgical planning. If MRI suggests bony defect, perform a low-dose thin-slice CT.
Ultrasound (high-frequency)	Bedside evaluation of superficial lesions (cystic vs. solid), assessment of vascularity, and guidance for aspiration/biopsy	High-frequency linear transducer (typically 7–15 MHz or higher), multiplanar scanning	Cannot penetrate intact bone—cannot evaluate intracranial components or tracts crossing intact skull base. Operator dependent (sensitivity/specificity vary); unreliable for deep tract mapping.	First-line, noninvasive bedside screening of palpable superficial cysts (helps avoid unnecessary radiation or anesthesia). Useful for guided aspiration or preoperative bedside assessment, but MRI/CT required if tract or intracranial extension is suspected.
Nasal endoscopy/intraoperative tract tracing	Direct visualization of external/internal openings, intraoperative confirmation of tract/residual tissue, and tract tracing (dyes/saline)	0°, 30°, 70° endoscopes; intraoperative tracers (dyes or saline)	Endoscopic view is limited by nasal anatomy and anterior nasal vestibule; cannot fully evaluate course posterior to cribriform plate or intracranial extension. Tracing may introduce bacteria into the tract, increasing infection risk; improper tracing can create false channels.	Useful to localize cutaneous or intranasal openings and to assist minimally invasive resections while preserving cosmesis. Does not replace comprehensive imaging evaluation.
Intraoperative navigation/intraop imaging (CT/MRI/ultrasound)	Improve resection accuracy, provide real-time or near-real-time localization, confirm complete resection, and assist in complex/recurrent/intracranial cases	Preoperative MRI/CT co-registration; when available, intraoperative low-dose CT or O-arm registration	High equipment and cost burden. Registration introduces errors (commonly sub-millimeter to ∼2 mm), which are affected by registration method and patient positioning—intraoperative verification against anatomical landmarks is mandatory. Misuse can create a false sense of precision.	Recommended as an adjunct for complex cases, recurrent disease, or lesions with intracranial extension when precise protection of nasal bone and soft-tissue structures is required.

MRI, magnetic resonance imaging; CT, computed tomography; US, ultrasound; STIR, short tau inversion recovery; O-arm, intraoperative CT system.

### 5.1 CT (computed tomography)

**Figure 2:**
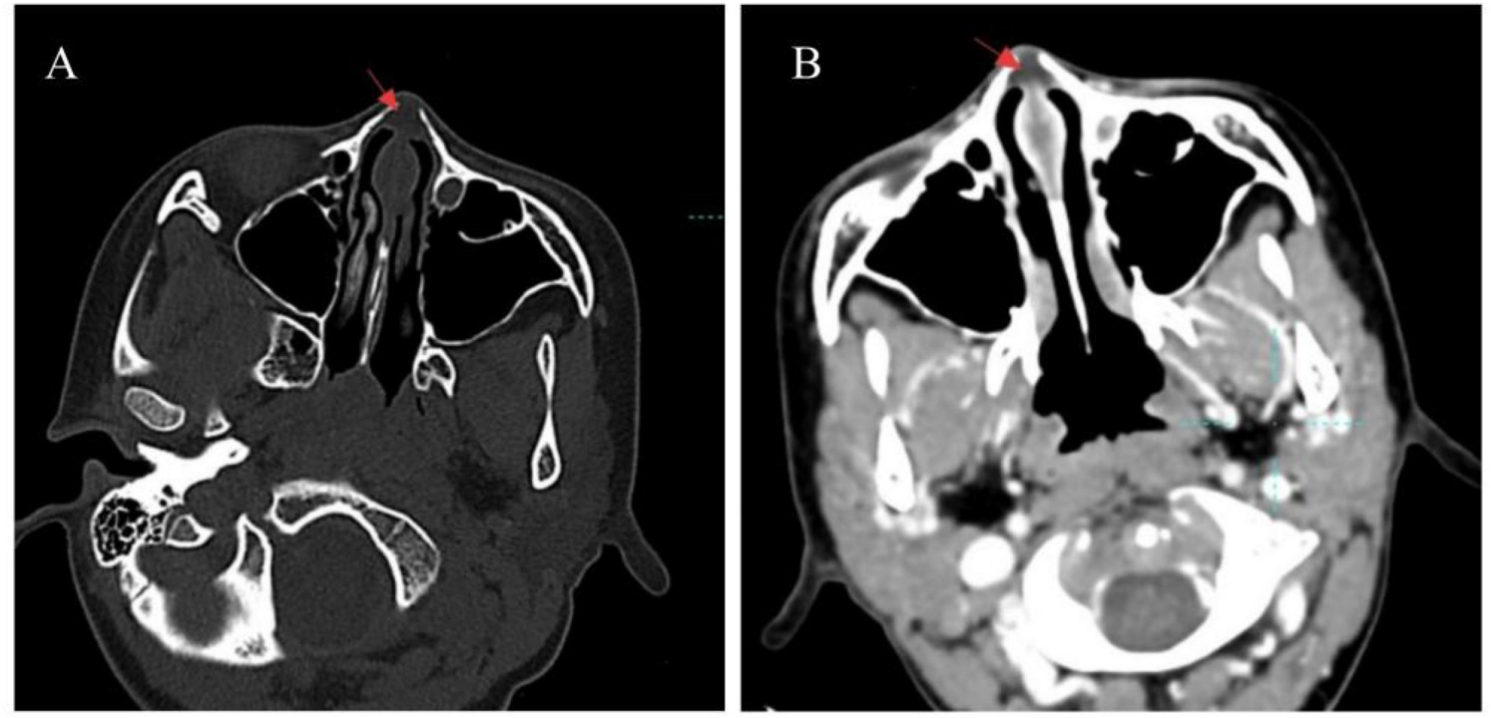
Representative CT image of nasal dermoid sinus cysts in pediatric. **(A)** A cystic low-density shadow in the middle of the nasal bone at the location indicated by the arrow, with a clear boundary. The bilateral nasal bones are compressed and displaced, presenting an arc-shaped indentation. **(B)** The area indicated by the arrow shows significant enhancement of the anterior soft tissue of the nasal septum after CT enhancement.

Computed tomography (CT) plays an important role in the evaluation of pediatric nasal dermoid/sinus cysts by allowing clinicians to define the extent of bony involvement and detect osseous defects (13). CT offers high spatial resolution for bone and is particularly advantageous in demonstrating intrabony lesions and whether a cyst traverses the nasal bones or breaches the skull base (17). In addition, CT provides relatively accurate delineation of the course of any fistulous tract and its relationship to surrounding anatomical structures, which assists in formulating targeted surgical approaches (13). However, CT has inherent limitations in soft-tissue contrast and may be insufficient to precisely discriminate the cyst from adjacent soft tissues or to fully characterize intracranial extension, thereby restricting its diagnostic utility in complex lesions or when intracranial involvement is suspected (7, 18). Moreover, CT exposes children to ionizing radiation; studies have reported associations between cumulative cranial CT radiation doses and increased risks of hematologic malignancies and brain tumors, so indications for CT in the pediatric population should be rigorously justified to avoid unnecessary exposure (19–21). Overall, while CT is a commonly used modality for assessing nasal dermoid/sinus cysts—offering clear advantages for bone imaging and tract localization—its limitations in soft-tissue resolution and radiation safety must be balanced. In clinical practice, the selection of CT should be tailored to the specific diagnostic question, and MRI should be used as a complementary modality when more detailed evaluation of soft tissues and intracranial structures is required. The representative CT images of nasal dermoid sinus cysts in Pediatric are shown in Figure 2.

### 5.2 MRI (magnetic resonance imaging)

**Figure 3:**
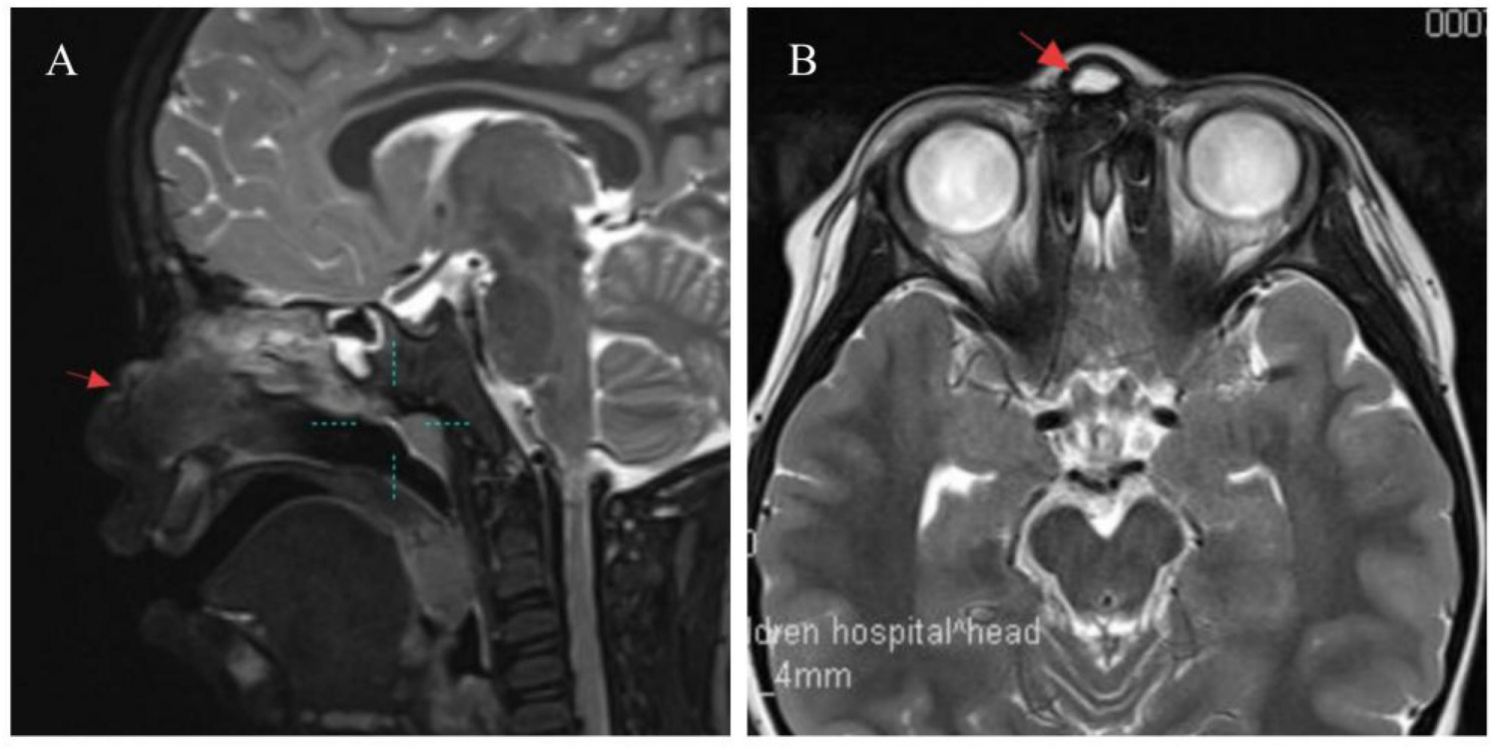
Representative MRI image of nasal dermoid sinus cyst in pediatric. **(A)** The area indicated by the arrow is a nodular long T1 and long T2 signal at the root of the nose. The mass is connected to the anterior skin and no communication with the intracranial cavity is seen. The posterior wall bone tissue is slightly compressed. **(B)** The tumor indicated by the arrow shows a high signal of T2 lipids and has a clear boundary.

Magnetic resonance imaging (MRI) is regarded as the imaging modality of choice for the evaluation of pediatric nasal dermoid/sinus cysts, offering a marked advantage in soft-tissue resolution (22). MRI can precisely depict the anatomical relationships between the cyst and surrounding soft tissues, thereby facilitating assessment of lesion margins and the impact on adjacent structures (23). For detecting meningeal or brain involvement, MRI demonstrates high sensitivity and specificity in distinguishing intracranial extension and defining the extent of disease; one study reported MRI sensitivity and specificity for intracranial extension of 100% and 95.7%, respectively, superior to CT (24). In pediatric patients, the non-ionizing nature of MRI is particularly important, as it avoids the cumulative radiation risk associated with repeated studies and is therefore better suited to children who require serial follow-up (22, 25). Commonly used sequences include T1-weighted, T2-weighted, contrast-enhanced and fat-suppression sequences; different sequences help to characterize the cyst contents and differentiate components such as fat and proteinaceous material (26). Moreover, advanced sequences such as diffusion-weighted imaging have demonstrated utility in the assessment of complex soft-tissue lesions (27). MRI supplies detailed lesion localization for preoperative evaluation and surgical planning, playing a pivotal role in promoting multidisciplinary collaboration and optimizing diagnostic and treatment pathways. The typical MRI image of nasal dermoid sinus cyst in Pediatric is shown in Figure 3.

### 5.3 Ultrasound (US) and endoscopy

Ultrasound is an important imaging tool for evaluating superficial lesions of pediatric nasal dermoid/sinus cysts and is widely used in children (28, 29). Ultrasound examination is noninvasive, convenient and can be performed at the bedside, making it especially appropriate for very young patients and reducing reliance on more advanced imaging modalities (28, 30). Technically, ultrasound can accurately demonstrate lesion location, size and internal structure of the cyst, and is effective for identifying scarring, the course of sinus tracts and soft-tissue abnormalities (30, 31). Clinical practice shows that the majority of cases can be assessed adequately by ultrasound, which has high sensitivity and, for some patients, may obviate the need for additional imaging (28). However, ultrasound has clear limitations in revealing deep complex tracts or intracranial extension, and it is difficult to accurately determine the relationship between the lesion and bony structures or the dura (24). Nasal endoscopy facilitates direct visualization of the nasal cavity and the opening of the tract within the anterior nares, providing necessary anatomic views for fistula localization and delineation of lesion extent (5, 13). Endoscopic assistance helps to delineate the course of the tract and the spatial relationship between the lesion and intranasal structures, thereby optimizing surgical approach and cosmetic outcomes (1, 5). Intraoperative endoscopic localization can improve the completeness and safety of excision, reduce morbidity associated with traditional techniques, and enhance postoperative cosmesis (5, 32). The combined application of ultrasound and endoscopy can optimize diagnostic and therapeutic pathways for nasal dermoid/sinus cysts and improve the efficiency of multidisciplinary management.

## 6 Surgical treatment strategies and techniques

Complete excision of the epithelial tract/cyst cavity to prevent recurrence and infection, while balancing aesthetic and functional reconstruction, is the core principle in the treatment of nasal dermoid/sinus cysts (17). Imaging plays an important role in preoperative evaluation: CT and MRI can delineate the lesion extent and guide the choice of surgical approach and safety assessment (4, 15), while the surgical method must be adapted to the lesion's extent and classification to achieve individualized management (13). Minimally invasive and endoscope-assisted approaches can improve surgical exposure, reduce complication rates, and at the same time preserve cosmetic outcomes (5, 17). To further decrease postoperative infection and complications, emphasis is placed on thorough removal of involved tissues and strict aseptic technique (33). Therefore, multidisciplinary team collaboration has a positive effect on managing complex cases and enhancing surgical safety; Table 3 summarizes the indications, advantages and disadvantages, and complications of commonly used surgical approaches.

**Table 3:** Comparison of common surgical approaches: indications, advantages, main risks/complications, and practical notes.

Surgical approach	Indications (by type/anatomy)	Advantages	Main risks/complications	Practical notes
Open rhinoplasty/external (facial) approach	Superficial or laterally placed nasal lesions without, or with only minimal, intracranial extension; cases requiring simultaneous aesthetic reconstruction or skin excision	Excellent exposure; facilitates complete excision of the cyst and tract entry; allows simultaneous cartilage/bony reconstruction to correct nasal-dorsum collapse and control cosmetic outcome	Facial scar (visibility depends on incision design), wound infection, sensory disturbance/traction injury to nerves, local flap perfusion problems, incomplete excision leading to recurrence	Mandatory preoperative MRI (soft-tissue/tract mapping) plus thin-slice CT (bone window) to rule out skull-base/cribriform defects. Control active infection before definitive surgery. Use endoscopic or surgical-microscope assistance intraoperatively to identify tract branches; if intracranial extension is suspected, be prepared for a combined approach. Meticulous incision design and layered closure optimize cosmetic outcome.
Endonasal endoscopic/endoscopic-assisted approach	Tracts or cysts that extend toward the nasal cavity, medially, or along the septum, and that do not have extensive intracranial/dural adherence	Minimally invasive with minimal external scarring and faster recovery; magnified view helps identification of the tract opening and local lesion; can be combined with other intranasal procedures	Inadequate exposure or limited visualization may result in incomplete excision and recurrence; risk of intranasal synechia, nasal bleeding or infection; unrecognized skull-base bony defects may lead to inadvertent dura breach and CSF leak	Preoperative MRI is required to exclude/assess intracranial extension (add thin-slice CT if indicated). Intraoperative navigation and combination of endoscope + microscope or dye tracing are recommended. If dural/brain adherence is encountered, convert promptly to a combined approach and consult neurosurgery. Endoscopic techniques are suitable for carefully selected minimally invasive cases or as endoscopic assistance to reduce external incisions.
Combined craniofacial/combined nasocranial approach	Confirmed intracranial extension (extradural/subdural/intradural or dural adherence), large cribriform/anterior skull-base defects, complex recurrent disease, or intraoperative discovery of adherence to brain/dura	Maximal exposure; direct management of skull-base defects and dural repair (reducing postoperative intracranial infection/recurrence); suitable when dural repair and reconstruction are required	Cerebrospinal fluid (CSF) leak, intracranial infection/meningitis, intraoperative hemorrhage, neurological injury, prolonged recovery, and prominent craniofacial scarring	Requires multidisciplinary team (ENT, neurosurgery, plastic surgery) consultation and preoperative high-resolution MRI + CT. Intraoperative use of autologous fascia, synthetic grafts or vascularized flaps is often necessary for dural and skull-base reconstruction. Consider short-term postoperative bedrest and selective lumbar drainage according to repair severity and risk; monitor closely for infection and intracranial complications. Literature suggests combined approaches reduce residual disease in intracranial cases but increase perioperative complexity and risk—these procedures should be performed in experienced centers.
Mini-open/hidden incision/percutaneous approach	Small, superficial lesions in patients with high cosmetic priority; imaging-confirmed absence of deep or skull-base extension	Small incision, less tissue trauma, hidden scar, shorter hospitalization and faster recovery	Limited exposure increases the risk of incomplete excision and recurrence; if deep extension is discovered intraoperatively, conversion to a wider approach is required (increasing the risk of reoperation)	Strict case selection required: preoperative MRI ± CT must confirm no deep or skull-base extension. If exposure is insufficient intraoperatively, promptly convert to a more extensive approach to ensure complete removal. Surgeons must inform families about the trade-off between cosmesis and completeness of excision and obtain written consent. Best suited for small, superficial lesions with short disease history and no recurrent infection.

### 6.1 External rhinoplasty approach

The external rhinoplasty approach is a well-established surgical route for the management of pediatric nasal dermoid/sinus cysts; it is appropriate for lesions located on the nasal dorsum or radix that have not extensively involved intracranial structures and where adequate exposure is required to ensure complete excision (1, 14). The principal advantage of this approach is that the external incision provides ample operative exposure and freedom of manipulation, facilitating protection of residual tissues and *en bloc* removal of the lesion in complex or anatomically indistinct cases, thereby minimizing residual disease and reducing the risk of recurrence (14). For lesions that extend deeply but do not involve the intracranial compartment, this route can also offer more reliable anatomic exposure to ensure margin integrity (34). Regarding aesthetic outcomes, studies have reported a low scar burden following external nasal incisions, high patient satisfaction with cosmesis, and noticeable improvement in facial contour (35, 36). When the nasal bone or soft tissues are compromised by the lesion or by surgery, cartilage grafting or other osseous reconstruction techniques are often employed to restore structural support and enhance postoperative appearance; long-term follow-up indicates that, provided complete excision is achieved, reconstruction can yield satisfactory functional and cosmetic results (17, 37). Nevertheless, individual variability may lead to subtle aesthetic sequelae such as pigmentary changes or mild soft-tissue depressions, which should be discussed with families preoperatively and incorporated into postoperative follow-up plans (38). Compared with a purely endoscopic approach, external rhinoplasty is associated with greater tissue trauma and a longer postoperative recovery, with increased needs for pain control, complication surveillance, and psychological support—particularly in pre-school children. For lesions that extend deeply but have not breached the skull base, the external rhinoplasty approach, in combination with endoscopic assistance when necessary, can expose the nasal dorsum and radix to permit more thorough lesion removal and reduce recurrence risk; therefore, for superficial lesions amenable to endoscopic or minimally invasive strategies, prioritizing minimally invasive or endoscope-assisted procedures can help lower the perioperative burden while balancing exposure and invasiveness (39, 40). Postoperative issues mainly include infection, local recurrence, and dissatisfaction with scarring or appearance (14, 35). Through comprehensive preoperative imaging assessment, meticulous microscopic/endoscopic-assisted excision, and multidisciplinary collaboration among otolaryngology, skull base surgery, and plastic surgery teams, the incidence of these complications can be minimized and reconstructive outcomes optimized (1).

### 6.2 Transnasal endoscopic approach

The transnasal endoscopic approach has become an important minimally invasive strategy for the removal of pediatric nasal dermoid/sinus cysts and their associated tract lesions, with clear indications for lesions confined to the nasal cavity, nasal septum, or superficial sinus tracts (13, 32), and has demonstrated favorable cure rates and postoperative complication profiles in carefully selected cases (41). Grounded in the principle of minimal trauma, this technique accesses the lesion via small intranasal incisions or natural lumina, minimizing disruption of bone and soft tissues; it achieves excellent cosmetic outcomes postoperatively and can substantially reduce patients' scar burden and associated morbidity (1, 32). Endoscope-assisted excision offers a magnified intraoperative view that facilitates meticulous dissection of tract termini and occult branches, thereby improving rates of complete resection and lowering the risk of recurrence (32). Moreover, several studies report that the endoscopic approach can maintain favorable nasal form and function on long-term follow-up, with stable recovery of respiration and olfaction, satisfactory aesthetic results, and no observed surgery-related complications or recurrence (38, 42). However, endoscopic techniques are not universally applicable: in cases with complex or marked intracranial extension, extensive bony destruction, or clearly evident cutaneous fistulae, the endoscopic field and working corridor may be limited, and combining an external rhinoplasty approach or other adjunctive measures should be considered to ensure surgical safety and completeness of lesion removal (1, 32, 41). Additionally, the combined use of imaging evaluation is of great importance for optimizing the surgical route and planning, guiding individualized approach selection and enhancing the thoroughness of resection (13). During the operation, protection of the nasal cavity and surrounding critical anatomical structures is essential for preventing and managing complications (43). For complex cases, multidisciplinary collaboration (e.g., combined otolaryngology and neurosurgery teams) can further improve surgical success rates and facilitate postoperative functional recovery (44, 45).

### 6.3 Combined intracranial–extracranial approach

The combined intracranial–extracranial approach is principally indicated for nasal dermoid/sinus cysts with intracranial extension or lesions that are adherent to the dura mater, particularly when the disease extends beyond the floor of the frontal sinus or forms complex sinus tracts (44, 46). These procedures typically require collaboration among a multidisciplinary team—otolaryngology, neurosurgery, and plastic/reconstructive surgery—to maximize surgical exposure while simultaneously addressing reconstructive and aesthetic outcomes (44, 47). Preoperative high-resolution MRI and CT imaging are employed to delineate lesion extent, evaluate intracranial involvement, and guide individualized surgical planning (13, 24). The operative plan should be developed jointly by the multidisciplinary team based on imaging findings, lesion complexity, and patient-specific anatomy, with combined approaches emphasized in high-risk or recurrent cases (48, 49). Close postoperative imaging follow-up is necessary to monitor for complications and recurrence (13). In current clinical decision-making, combined approaches offer clear advantages for lesions tightly adherent to the dura and for deep sinus tracts, facilitating complete resection of the lesion and its tracts and reducing the risk of bony recurrence (46, 47). For patients with prior frontal sinus opening and reconstruction, a combined endoscopic and craniotomy approach may lower early and long-term complication rates (50). However, indications for the combined intracranial–extracranial approach remain controversial. Some studies advocate a cranial combined approach for all cases with intracranial involvement to ensure thorough excision and minimize recurrence (51, 52), whereas others emphasize that for mild intracranial extension, isolated endonasal or external approaches may be preferable with regard to cosmesis and morbidity (1, 8). Therefore, selection of the surgical route should integrate lesion anatomy, radiological assessment, and the patient's postoperative aesthetic needs, and be decided collaboratively by the multidisciplinary team.

### 6.4 Other approaches

In addition to the conventional external rhinoplasty and endoscopic transnasal routes, some cases may be managed by lesion excision through a small subcutaneous incision, a longitudinal midline incision, or specially combined approaches (5, 13). A small subcutaneous incision is primarily indicated for lesions confined to the superficial tissues without deep or osseous involvement; by creating a linear skin incision directly over the lesion, the cyst can be dissected and removed intact, yielding favorable wound healing and minimal trauma (53). The longitudinal midline approach is suitable for lesions involving the nasal bone or when intracranial extension is suspected; this technique uses a longitudinal midline incision combined with endoscopic assistance to excise the cyst and can provide adequate exposure for thorough removal in anatomically complex regions (13). Furthermore, reports describe lesions located in other complex anatomical areas—such as those adjacent to the orbit or the forehead—that may be addressed using subbrow or forehead-combined incisions when appropriate (53). However, smaller or specialized incisions commonly limit surgical exposure, which increases the risk of residual disease and recurrence; operations in these special anatomical regions are technically demanding and place higher requirements on the surgeon (5, 53). Complications are mainly local infection, scarring, and recurrence, and their incidence varies with the chosen approach and the complexity of the lesion (13). Therefore, special approaches must be selected prudently according to lesion extent, patient anatomy, and cosmetic considerations, and should rely on multidisciplinary team decision-making to optimize efficacy and safety (45).

## 7 Age-related differences in diagnosis and treatment

The diagnosis and management of pediatric nasal dermoid/sinus cysts should be individualized within a multidisciplinary framework (otolaryngology, neurosurgery, and plastic/reconstructive surgery) according to age-related anatomy, the child's ability to cooperate with imaging, and perioperative tolerance (38, 54). Neonates and infants (0–2 years) often present with superficial findings noted by caregivers and may develop rapid suppuration when infected; any suspicious cutaneous change should prompt MRI as the first-line study to exclude intracranial extension (MRI delineates soft-tissue relationships to the cranial cavity without ionizing radiation), although for children under 5 years the risks of MR sedation/anesthesia must be balanced with the anesthesia team; bedside ultrasound can serve as an initial rapid screen, and when assessment of bone detail or preoperative osseous windows is necessary a low-dose CT may be obtained after careful justification and using pediatric low-dose protocols (2, 13, 17, 51, 55). In the preschool to early school-age period (3–6 years), improved cooperation allows most children to complete MRI without deep sedation, but discrepancies between imaging and intraoperative findings can occur and imaging should be interpreted together with clinical signs; patients with a history of recurrent infection or prior incision and drainage frequently have scar adhesion encountered intraoperatively, often necessitating wider exposure and thorough debridement with readiness for dural/anterior cranial base repair; when infection is controlled preoperatively and procedures are performed by an experienced multidisciplinary team (MDT), increased surgical complexity can still yield satisfactory long-term outcomes and does not necessarily translate into a higher recurrence rate (38, 56, 57). In older school-age children and adolescents (≥7 years), aesthetic concerns and greater tissue tolerance favor preoperative high-resolution MRI (with CT bone windows if indicated) to precisely delineate the lesion and anterior cranial base anatomy; treatment commonly employs combined approaches (external rhinoplasty ± endoscopic, or combined extra-cranial and intracranial approaches), and simultaneous or staged structural reconstruction (autologous cartilage/fat/bone grafting, etc.) is often performed to optimize both functional and cosmetic results (17, 55, 58, 59). At any age, clinical or radiological signs suggesting dural or intracerebral involvement (e.g., CSF-like discharge, deep fistulae, or imaging evidence of transcranial extension) should trigger an immediate MRI and early neurosurgical consultation; cases with intracranial extension typically require combined ENT–neurosurgery–plastic surgery approaches, meticulous intraoperative dural repair and anterior cranial base reconstruction, and intensified postoperative antimicrobial therapy with imaging follow-up to reduce the risk of major complications and recurrence (51, 56, 60). Regarding imaging and perioperative management, recent evidence and institutional experience recommend MRI as the routine first-line modality, with ultrasound used for rapid bedside screening; CT should only be performed when there is a clear need to evaluate bony structures or when MRI cannot be obtained in a timely manner, and strict adherence to pediatric lowest-dose principles is required; for young children who need sedation, needle-free administration regimens exemplified by intranasal dexmedetomidine, or combinations with low-dose propofol/midazolam, may be preferred to balance image quality with anesthetic safety and to facilitate cooperation (61–64). Regarding radiation risk, studies have suggested an association between cumulative bone-marrow dose from pediatric CT and the risk of hematologic malignancies, so CT indications in children should be tightly controlled and doses optimized—this requires the clinical team to carefully weigh diagnostic benefit against radiation risk (17, 19). Recurrence is most often due to incomplete excision or involvement of fistulous tracts/cranial base, and age is not the sole determining factor; an individualized follow-up plan of at least 1–3 years (clinical assessment ± imaging as necessary) should be established according to lesion complexity and surgical approach, and MDT management is recommended for high-risk cases or those with intracranial extension to improve safety and long-term outcomes (2, 38, 57, 59).

## 8 Perioperative management

In perioperative management, preoperative infection control warrants careful attention because local infection not only increases the risk of postoperative complications and recurrence, but surgery should also be avoided during acute infectious episodes (35, 65). Postoperative care should prioritize incision care and infection surveillance (13, 66). Preoperative review of imaging is crucial for delineating the extent and localization of the cyst, determining the presence of intracranial extension, and formulating an appropriate surgical plan (4, 13); local recurrence and postoperative infection are frequently associated with incomplete preoperative imaging assessment (35). Moreover, individualized anesthesia and perioperative supportive measures have demonstrated beneficial effects in safeguarding the child's safety, reducing adverse events, and improving adherence (67). Postoperative follow-up durations are typically concentrated from one to several years, and some studies recommend scheduled imaging surveillance to monitor for recurrence and nasal development (13, 17); given that recurrence is often attributable to incomplete excision or case complexity, long-term follow-up facilitates early detection (14, 59). Therefore, emphasizing standardized management, multidisciplinary team involvement, and prolonged follow-up is of considerable importance for improving prognosis.

## 9 Multidisciplinary (MDT) management pathway

**Figure 4:**
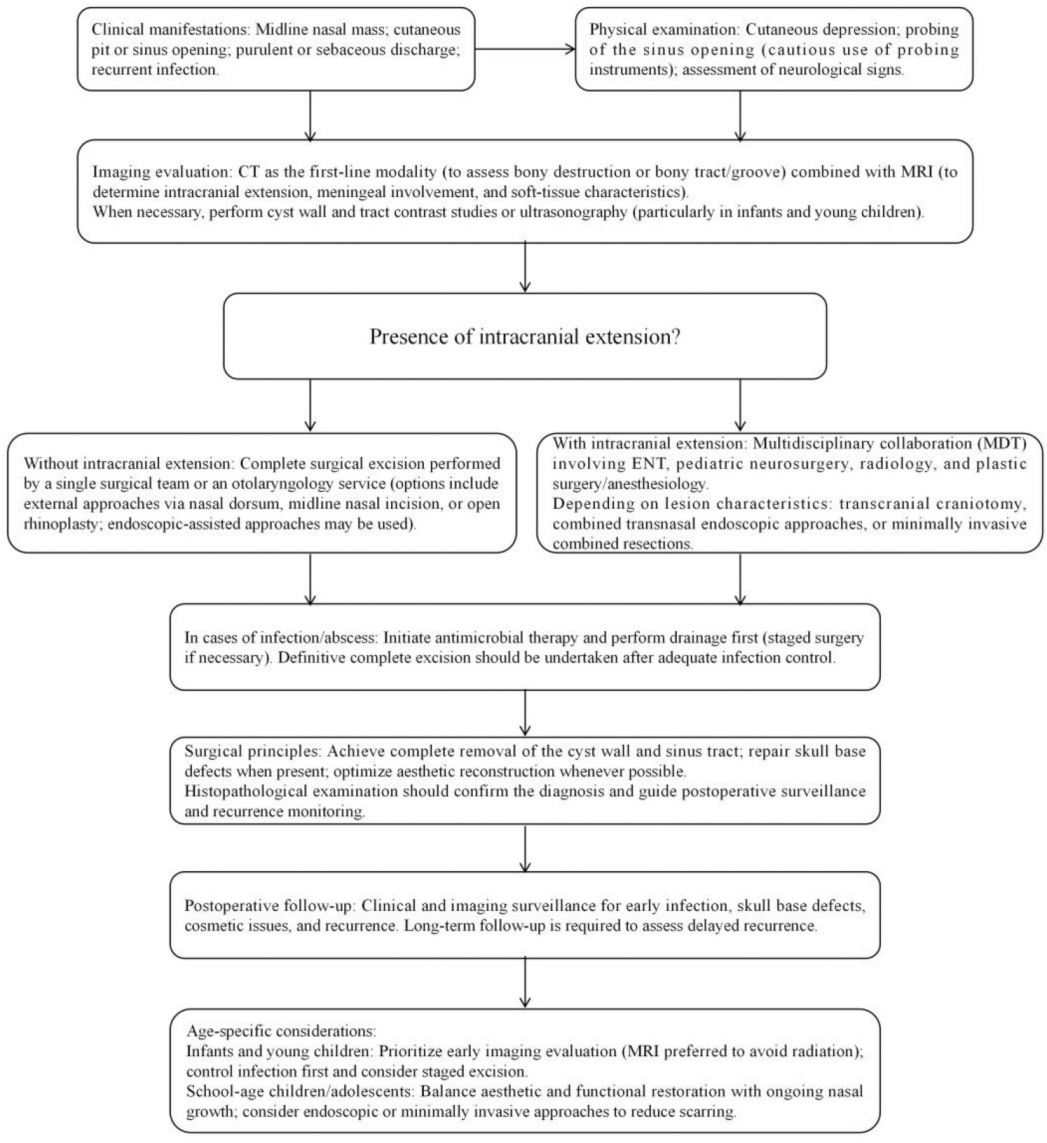
Flowchart of MDT diagnosis and management.

Multidisciplinary team (MDT) management plays a pivotal role in the diagnosis and treatment of pediatric nasal dermoid/sinus cysts, particularly when lesions involve complex anatomical structures or demonstrate intracranial extension (32, 44). The MDT model integrates core specialties such as pediatrics, otorhinolaryngology, and neurosurgery and is essential for jointly formulating comprehensive diagnostic and therapeutic plans (44), Table 4 and Figure 4 summarized the responsibilities of MDT members and the consultation path. In cases with intracranial involvement, neurosurgery is responsible for exposure and resection of deep-seated lesions, whereas plastic surgery focuses on repair of nasal and craniofacial structures and aesthetic reconstruction (44, 68). Otolaryngology typically leads preoperative assessment and lesion resection, coordinating surgical planning and perioperative management (17, 32). Radiology provides precise lesion localization and topographic analysis via CT and MRI, assisting in differential diagnosis and in delineating the extent of intracranial extension (7, 24). Pathology contributes to postoperative confirmation and recurrence-risk assessment; in complex cases, pathological findings guide subsequent management and long-term follow-up (17). Plastic surgery's intraoperative aesthetic and structural repairs achieve both functional and cosmetic improvements, substantially enhancing parental confidence and satisfaction (68, 69). The anesthesia team participates in perioperative management of complex cases, supporting safe pediatric anesthesia and intraoperative monitoring, while nursing and rehabilitation teams concentrate on postoperative surveillance, wound care, and functional recovery (70, 71). The development of standardized MDT case-discussion workflows emphasizes individualized imaging-based assessment, joint multidisciplinary decision-making, and coordinated execution of surgery to ensure resection completeness and complication prevention (72, 73). In clinical practice, multidisciplinary pathways implement continuous collaboration across preoperative assessment, surgical intervention, and postoperative follow-up, and they are particularly valuable in improving efficacy and reducing recurrence in complex or high-risk cases (32, 44). Collaborative management not only optimizes the surgical process but also promotes cosmetic and functional recovery, thereby improving patient satisfaction and long-term outcomes (17, 68). However, the recommended strength and scope of the MDT model vary between centers and lack unified standards. Therefore, we suggest that multidisciplinary management pathways be graded according to the GRADE (Grading of Recommendations, Assessment, Development and Evaluation) framework. For complex cases with intracranial extension, combined multidisciplinary surgery may be classified as a “strong recommendation,” whereas for superficial lesions confined to the nasal region, single-specialty-led surgical management may be considered a “conditional recommendation.” Introducing the GRADE framework would help reinforce the standardization and transparency of recommendations and provide clearer evidence-based guidance for clinical practice.

**Table 4:** MDT roles and consultation pathway.

Team	Pre-operative role	Intra-operative role	Post-operative role	Decision trigger/notes
ENT/Head & Neck Surgery	History and physical examination; local nasal/external nasal assessment; formulate preliminary surgical approach and excision plan; counsel family on complications and cosmetic expectations	Responsible for primary exposure and excision of the lesion (mainly external nasal or endonasal disease); coordinate with neurosurgery/plastic surgery for margin management	Wound care, nasal cavity care, outpatient follow-up; coordinate with nursing and rehabilitation	ENT leads when imaging shows localized intranasal or external nasal disease without clear intracranial extension; consult neurosurgery if skull-base or dural involvement is suspected
Neurosurgery	Assess for intracranial extension and risk of dural/brain adhesion; participate in discussion of need for craniotomy/dural repair	Perform intracranial exposure, dural resection and repair, and skull-base reconstruction; operate synchronously with ENT/plastic surgery as required	Monitor for neurological complications, manage CSF leak, imaging follow-up and long-term neurological assessment	Mandatory involvement when MRI/CT shows intracranial extension, dural involvement, skull-base bone defects, or in the setting of prior recurrence/infection
Plastic Surgery	Evaluate need for facial reconstruction; design cosmetic incisions; prepare donor sites (cartilage, fascia)	Participate in incision and flap design and reconstruction; implant cartilage/bone materials and restore facial contour	Assess cosmetic outcome, manage scarring, perform secondary refinements	Involved when there is significant soft-tissue loss, need for reconstruction, or strong family demand for cosmetic optimization; join intraoperatively if large resection is performed
Radiology	Interpret high-resolution MRI (preferred) and CT (bone window); provide 3D reconstructions and navigation datasets	Provide intraoperative navigation support or intraoperative imaging (ultrasound/fluoroscopy) as available	Postoperative imaging surveillance and radiologic assessment of complications (fluid collections, recurrence)	Radiology consult is required when MRI suggests intracranial extension or CT demonstrates skull-base defects/complex 3D relationships; provide registered datasets for intraoperative navigation
Pathology	Advise on biopsy/aspiration indications and preoperative diagnostic expectations	Provide intraoperative frozen section assessment for margins/presence of epithelial remnants; give rapid feedback on pathological findings	Deliver formal paraffin pathology report and recommend further management for malignant or atypical findings	Intraoperative frozen section is triggered when confirmation of complete excision or epithelial remnant is required; microbiology advised if infection/inflammation is suspected
Anesthesiology	Assess anesthetic risk (pediatric weight, airway, comorbidities); plan anesthesia and hemodynamic management	Manage anesthesia maintenance, airway, temperature and fluid balance; handle intraoperative emergencies	Postoperative emergence, analgesia, and monitoring for complications (respiratory compromise, bleeding)	Recommend specific perioperative strategies for difficult pediatric airway, prolonged procedures, or intracranial operations (positioning/intracranial pressure considerations)
Nursing & Rehabilitation	Preoperative education and preparation (skin cleansing, prophylactic antibiotics, family instruction)	Sterile instrument and nursing assistance, intraoperative documentation, immediate postoperative transfer	Wound and nasal care, rehabilitative training (nasal function, scar rehabilitation), discharge planning and follow-up	Individualized care plans for patients with infection history or anticipated long-term nasal function/scar rehabilitation needs
Pediatrics/Neonatology	Comprehensive pediatric assessment (development, nutrition, comorbidities); plan perioperative pediatric support	Perioperative physiological management and handling of pediatric complications	Growth and development follow-up; vaccination and nutrition guidance	Required involvement for preterm infants, low birth-weight infants, or children with significant cardiopulmonary or systemic disease

## 10 Clinical translation

In recent years, translational research on pediatric nasal dermoid/sinus cysts has displayed a trend toward multidisciplinary collaboration that accelerates innovation in diagnostic and therapeutic paradigms (5, 32). In the realm of molecular biomarkers, direct reports specifically addressing NDSC remain absent; related investigations have concentrated on microRNAs as early diagnostic and risk-prediction tools in other pediatric neoplasms, yet no practical application in the field of nasal dermoid/sinus cysts has been reported (74). Human molecular developmental studies indicate that perturbations—either genetic mutations or dysregulated expression—in key regulators such as ALX1, SIX2, SHH (Sonic Hedgehog) and FGF (Fibroblast Growth Factor) can impair frontonasal mesenchymal development and precipitate midline craniofacial anomalies and fissures (9, 75, 76). Notably, the pivotal role of the SHH signaling pathway in formation of midline structures has been confirmed; its aberrant activation or suppression may cause midline fusion defects during embryogenesis (77), whereas dysregulation of FGF signaling may further aggravate midline abnormalities by altering cellular proliferation and differentiation processes (78). Although direct evidence linking these genetic abnormalities specifically to nasal dermoid/sinus cysts is currently lacking, the aforementioned molecular mechanisms offer important clues for further mechanistic exploration and underscore the need for more in-depth study.

Furthermore, the advent of single-cell sequencing technologies provides a novel perspective for interrogating cellular heterogeneity within complex tissues, and their application to craniofacial developmental disorders is receiving increasing attention (79, 80). Single-cell RNA sequencing (scRNA-seq) enables dissection of the transcriptomic signatures of midline tissue cells during embryogenesis, thereby uncovering critical molecular drivers (80). The combination of genomics and proteomics approaches—such as mass spectrometry (MS) and CRISPR screening—also holds promise for identifying potential therapeutic targets associated with nasal dermoid/sinus cysts (81, 82). Radiomics, as an emerging analytic methodology, can extract multidimensional features from large imaging datasets to support precise subtyping, risk assessment, and prognostication; its application in the NDSC domain remains exploratory but carries substantial potential (83).

Regarding embryological mechanisms, multiple studies indicate that cyst formation is closely associated with fusion defects of midline structures, and histopathological classification schemes have provided a theoretical foundation for refining clinical diagnostic protocols, yet the underlying molecular pathological mechanisms remain unclear (3, 6). From a surgical-technical perspective, intraoperative navigation is progressively being incorporated into endoscopic practice; by enhancing operative safety, localization accuracy, and surgeon ergonomics, it has improved the feasibility of resecting complex lesions (84, 85). With iterative advances in imaging technology, the combined application of CT and MRI has become the standard approach for assessing lesion extent and guiding surgical trajectories; notably in young children, MRI's superior soft-tissue resolution has increased the detection rate of intracranial extension (1, 7). As an emerging analytic method, radiomics can extract multidimensional features from large-scale imaging datasets to support precise subtyping, risk stratification, and prognostic prediction; its use in prostate cancer and ovarian dermoid cysts has demonstrated diagnostic performance comparable to that of senior radiologists, but its role in the NDSC field remains exploratory (86, 89). Endoscopic minimally invasive techniques are increasingly the mainstay of surgical treatment for NDSC, and the literature shows that transnasal endoscopic approaches or combined approaches with external rhinoplasty can reduce soft-tissue injury, shorten incision length, and improve cosmetic outcomes while maintaining low complication rates (1, 5). In optimizing aesthetic incision strategies, unilateral longitudinal incisions or combined surgical pathways supplemented by minimally invasive techniques may improve aesthetic satisfaction and reduce scar burden, although specific tactics require long-term follow-up to assess their effectiveness (13, 32). Multidisciplinary teams have driven the standardization of diagnostic and therapeutic workflows; collaboration among surgical, radiological, and neurosurgical specialties ensures scientifically grounded surgical planning and safe execution, and plays a critical role in facial reconstruction and management of complex cases (32, 88).

## 11 Conclusion

This review, conducted within a multidisciplinary team (MDT) framework, systematically summarizes the embryologic mechanisms, imaging assessment, surgical strategies, and advances in clinical translation for pediatric nasal dermoid sinus cyst (NDSC), and it delineates the strengths and limitations of the current evidence base. Existing studies support abnormal midline fusion and ectodermal inclusion as the primary pathogenic mechanisms. Imaging-based classification plays a central role in risk stratification and in selecting the surgical approach; magnetic resonance imaging (MRI) is the preferred modality, while computed tomography (CT), ultrasound, and endoscopy provide complementary value for assessment of bony anatomy, bedside screening, and intraoperative localization. Complete excision of the sinus tract/cyst remains the key to reducing recurrence, whereas endoscopic minimally invasive techniques, combined surgical approaches, and intraoperative navigation are improving exposure safety and cosmetic outcomes. It should be emphasized that current evidence derives mainly from case series and retrospective cohort studies and is commonly limited by small sample sizes, heterogeneous standards, and insufficient follow-up, resulting in overall evidence levels that are low to moderate; consequently, only conditional recommendations can be made for most treatment options. By contrast, combined MDT evaluation and combined surgical approaches receive a stronger recommendation for high-risk cases such as those with intracranial extension. Future research urgently requires multicenter prospective cohorts, standardized imaging and surgical outcome metrics, long-term follow-up, and mechanistic investigations integrating molecular omics and radiomics to improve evidence quality, optimize risk stratification, and facilitate the development of stronger, more generalizable clinical guidelines. In summary, management of NDSC should adhere to imaging-driven individualized decision-making, MDT collaboration, and standardized follow-up in order to ensure complete resection while also safeguarding children's long-term cosmetic and functional outcomes.

## References

[1] Mostafa K Mostafa R Nezam S Nezam N Shaheen F. Surgical approach to congenital nasal dermoid sinus cyst in adult with external rhinoplasty and endoscopic approach: a case report. Ann Med Surg. (2024) 86(10):6153–58. 10.1097/ms9.0000000000002441 PMC11444641 39359779

[2] Naina P Jonathan GE Prabhakar M Irodi A Syed KA John M Pediatric nasal dermoid- a decade’s experience from a south Indian tertiary care centre. Int J Pediatr Otorhinolaryngol. (2020) 139:110418. 10.1016/j.ijporl.2020.110418 33035807

[3] Zheng Y Ye B Lv J Xiang M. Progress of the diagnosis and treatment of congenital nasal dermoid sinus cysts. J Clin Otorhinolaryngol Head Neck Surg. (2021) 35(2):176–80. 10.13201/j.issn.2096-7993.2021.02.020 PMC10127880 33541005

[4] Phelan AL Jones CM Ceschini AS Henry CR Mackay DR Samson TD. Sparing a craniotomy: the role of intraoperative methylene blue in management of midline dermoid cysts. Plast Reconstr Surg. (2017) 139(6):1445–51. 10.1097/prs.0000000000003369 28538574

[5] Lietaer C Hens G Van Gerven L. Endoscopic-assisted removal of a nasofrontal dermoid cyst with intracranial extradural extension. Eur Arch Otorhinolaryngol. (2025) 282(5):2775–80. 10.1007/s00405-025-09249-4 39937276

[6] Kotowski M Szydlowski J. Congenital midline upper lip sinuses with intracranial extension – a variant of nasal dermoid? An embryology-based concept. Int J Pediatr Otorhinolaryngol. (2022) 164:111394. 10.1016/j.ijporl.2022.111394 36459726

[7] Kotowski M Szydlowski J. Radiological diagnostics in nasal dermoids: pitfalls, predictive values and literature analysis. Int J Pediatr Otorhinolaryngol. (2021) 149:110842. 10.1016/j.ijporl.2021.110842 34329830

[8] Kohan J McGee SA Self Q Ahern T Hersey D Malley O Operative options for extracranial nasal dermoid cysts: a meta-analysis. J Plast Reconstr Aesthet Surg. (2024) 88:171–81. 10.1016/j.bjps.2023.10.114 37983980

[9] Iyyanar PPR Wu Z Lan Y Hu Y Jiang R. Alx1 deficient mice recapitulate craniofacial phenotype and reveal developmental basis of alx1-related frontonasal dysplasia. Front Cell Dev Biol. (2022) 10:777887. 10.3389/fcell.2022.777887 35127681 PMC8815032

[10] Hufnagel RB Zimmerman SL Krueger LA Bender PL Ahmed ZM Saal HM. A new frontonasal dysplasia syndrome associated with deletion of the six2 gene. Am J Med Genet A. (2015) 170(2):487–91. 10.1002/ajmg.a.37441 PMC8108007 26581443

[11] Kuzu S. Broad, recurrent nasal dermoid cyst. J Craniofac Surg. (2020) 31(8):e760–61. 10.1097/scs.0000000000006683 33136903

[12] Barbour A Penman D Kubba H. Prevalence of thyroid gland tissue in midline neck dermoid cysts in children and a proposed new ‘thyroglossal entrainment’ hypothesis for their formation. J Laryngol Otol. (2023) 138(4):448–50. 10.1017/s0022215123001792 37795741

[13] Ni K Li X Zhao L Wu J Liu X Shi H. Diagnosis and treatment of congenital nasal dermoid and sinus cysts in 11 infants: a consort compliant study. Medicine. (2020) 99:e19435. 10.1097/md.0000000000019435 32481248 PMC7249947

[14] Makhdoom N Abo El Ezz TA Abdel-Haleem M. Management of midline nasal dermoid lesions in children by external rhinoplasty. J Taibah Univ Med Sci. (2017) 12(4):324–28. 10.1016/j.jtumed.2017.02.003 31435258 PMC6695084

[15] Molodtsova EV Yunusov AS Daikhes NA Polyakov DP Korobkin AS Larina OM. Our experience in the treatment of congenital nasal median heterotopias in children and an overview of various treatment tactics. Vestn Otorinolaringol. (2024) 89(2):28–32. 10.17116/otorino20248902128 38805460

[16] Ray CN Betteridge BC Demke JC. Infected nasal dermoid cyst/sinus tract presenting with bilateral subperiosteal supraorbital abscesses: the midline nasal tuft of hair, an overlooked finding. Ophthalmic Plast Reconstr Surg. (2017) 34(1):e31–34. 10.1097/iop.0000000000001000 29068832

[17] Carroll WW Farhood Z White DR Patel KG. Nasal dorsum reconstruction after pediatric nasal dermoid excision. Int J Pediatr Otorhinolaryngol. (2020) 140:110502. 10.1016/j.ijporl.2020.110502 33248715

[18] Kaminsky J Bienert-Zeit A Hellige M Rohn K Ohnesorge B. Comparison of image quality and in vivo appearance of the normal equine nasal cavities and paranasal sinuses in computed tomography and high field (3.0 t) magnetic resonance imaging. BMC Vet Res. (2016) 12(1):13. 10.1186/s12917-016-0643-6 26786270 PMC4717646

[19] Bosch De Basea M Thierry-Chef I Harbron R Hauptmann M Byrnes G Bernier M Risk of hematological malignancies from CT radiation exposure in children, adolescents and young adults. Nat Med. (2023) 29(12):3111–19. 10.1038/s41591-023-02620-0 37946058 PMC10719096

[20] Hauptmann M Byrnes G Cardis E Bernier M Blettner M Dabin J Brain cancer after radiation exposure from CT examinations of children and young adults: results from the epi-ct cohort study. Lancet Oncol. (2022) 24(1):45–53. 10.1016/s1470-2045(22)00655-6 36493793

[21] Lee S Kim HY Lee KH Cho J Lee C Kim KP Risk of hematologic malignant neoplasms from head ct radiation in children and adolescents presenting with minor head trauma: a nationwide population-based cohort study. Eur Radiol. (2024) 34(9):5934–43. 10.1007/s00330-024-10646-2 38358528

[22] Reddy A Kreicher KL Patel NA Schantz S Shinhar S. Pediatric epidermoid cysts masquerading as ranulas: a case series. Int J Pediatr Otorhinolaryngol. (2016) 81:26–8. 10.1016/j.ijporl.2015.11.031 26810284

[23] Ahlawat S Fritz J Morris CD Fayad LM. Magnetic resonance imaging biomarkers in musculoskeletal soft tissue tumors: review of conventional features and focus on nonmorphologic imaging. J Magn Reson Imaging. (2019) 50(1):11–27. 10.1002/jmri.26659 30793420

[24] Meira Pazelli A Wang L Gates-Tanzer L Davis DMR Cofer S Mardini S Imaging yield and surgical outcomes of nasal, medial brow, forehead, and scalp dermoid cysts. Cleft Palate Craniofac J. (2024). 10.1177/10556656241295572 39491820

[25] Zens TJ Rogers AP Riedesel EL Leys CM Ostlie DJ Woods MA The cost effectiveness and utility of a “quick MRI” for the evaluation of intra-abdominal abscess after acute appendicitis in the pediatric patient population. J Pediatr Surg. (2018) 53(6):1168–74. 10.1016/j.jpedsurg.2018.02.078 29673611

[26] Otonari-Yamamoto M Nakajima K Sakamoto J Imoto K Watanabe M Kotaki S Atypical MRI and histopathological findings in dermoid cyst. Bull Tokyo Dent Coll. (2018) 59(3):207–12. 10.2209/tdcpublication.2017-0044 30224615

[27] Abdel Razek AAK Sherif FM. Differentiation of sublingual thyroglossal duct cyst from midline dermoid cyst with diffusion weighted imaging. Int J Pediatr Otorhinolaryngol. (2019) 126:109623. 10.1016/j.ijporl.2019.109623 31400658

[28] Alsalek S Christian EA Esfahani DR. Ultrasound as a standalone tool for the management of pediatric calvarial dermoid cysts. Childs Nerv Syst. (2024) 40(12):4179–87. 10.1007/s00381-024-06521-6 38951208

[29] Ha D Kim T Shin K Kim H Kim B Kim M Ultrasonographic findings of pediatric dermoid cyst. Pediatr Int. (2021) 63(4):436–41. 10.1111/ped.14408 33576109

[30] Garcia C Wortsman X Bazaes-Nuñez D Pelizzari M Gonzalez S Cossio M Skin sonography in children: a review. Pediatr Radiol. (2022) 52(9):1687–705. 10.1007/s00247-022-05434-3 35821441

[31] Adekanmi A Adenigba P Lawal T Elemile P Onakpoma F. The value of ultrasonography in the diagnosis of a rare congenital dermoid cyst of the anterior fontanelle in an infant. J West Afr Coll Surg. (2022) 9(4):21–5. 10.4103/jwas.jwas_34_21 PMC9063536 35514791

[32] Woodyard De Brito KC Dembinski DR Lawera NG Buller M de Alarcon A Pan BS Transnasal endoscopic approach for excision of intracranial nasal dermoid sinus cysts. J Craniofac Surg. (2024) 36(1):30–6. 10.1097/scs.0000000000010582 39254437

[33] Kalmar CL Patel VA Taylor JA. Analysis of national outcomes for simple versus complex nasal dermoid cyst excision. J Craniofac Surg. (2020) 32(3):e281–83. 10.1097/scs.0000000000007160 33278252

[34] Locke R Kubba H. The external rhinoplasty approach for congenital nasal lesions in children. Int J Pediatr Otorhinolaryngol. (2011) 75(3):337–41. 10.1016/j.ijporl.2010.11.025 21183230

[35] Hori YS Albanese JS Meara JG Proctor MR. Occult intraosseous dermoid cyst at the nasofrontal junction. Pediatr Neurosurg. (2022) 58(1):58–60. 10.1159/000528440 36450236 PMC10064384

[36] Rand D Dlouhy BJ Kanotra SP. Extended external rhinoplasty approach for nasal dermoids with intracranial extension. Laryngoscope. (2023) 133(10):2798–802. 10.1002/lary.30551 36688249

[37] Aliyeva A. Novel surgical approach to nasal dorsum midline dermoid sinus cyst. Case Reports Plast Surg Hand Surg. (2023) 10(1):2256398. 10.1080/23320885.2023.2256398 37706218 PMC10496523

[38] Kotowski M Adamczyk P Szydlowski J. Nasal dermoids in children: factors influencing the distant result. Indian J Otolaryngol Head Neck Surg. (2022) 74(2):1412–19. 10.1007/s12070-021-02568-y 36452575 PMC9702314

[39] Seidel DU Sesterhenn AM. Intracranial nasal dermoid sinus cyst: transnasal endoscopic resection by open rhinoplasty approach, with intraoperative video. J Craniofac Surg. (2016) 27(8):2110–227. 10.1097/SCS.0000000000003107 28005764

[40] David AP House AE Chan DK. Endoscope-assisted open rhinoplasty approach for removal of nasal dermoid with intracranial extension. Otolaryngology Case Rep. (2020) 17:100211. 10.1016/j.xocr.2020.100211

[41] Vaz-Guimaraes F Koutourousiou M de Almeida JR Tyler-Kabara EC Fernandez-Miranda JC Wang EW Endoscopic endonasal surgery for epidermoid and dermoid cysts: a 10-year experience. J Neurosurg. (2019) 130(2):368–78. 10.3171/2017.7.JNS162783 29547084

[42] Xu H Li W Zhang H Wang H Hu L Wang D. Endoscopic endonasal surgery for dermoid cysts arising from the middle cranial fossa floor: a rare case series. Ear Nose Throat J. (2022) 104(9):NP577–86. 10.1177/01455613221138209 36380481

[43] Lu X Hang W Liu H Xue K Zhang X Liu G. Experience in the diagnosis and treatment of the postoperative complications of craniopharyngiomas through expanded endoscopic endonasal transsphenoidal approach. J Clin Otorhinolaryngol Head Neck Surg. (2021) 35(6):505–10. 10.13201/j.issn.2096-7993.2021.06.005 PMC10128607 34304508

[44] Davis MJ Abu-Ghname A Davies LW Xue AS Masoumy M Lam S Midline intranasal dermoid cyst with intracranial extension: technical details of a multidisciplinary approach to a rare condition. J Craniofac Surg. (2020) 31(3):e241–44. 10.1097/scs.0000000000006184 31985595

[45] Hoffmann TK Scheithauer MO Sommer F Lindemann J Haberl E Friebe-Hoffmann U Surgery of anterior skull base lesions in children. Ann Otol Rhinol Laryngol. (2017) 126(3):245–53. 10.1177/0003489416685321 28092970

[46] Shimizu R Sakamoto Y Miwa T. The necessity of dural resection for nasal dermal sinus cyst with intracranial extension. J Craniofac Surg. (2023) 34(6):e589–90. 10.1097/scs.0000000000009480 37336477

[47] Li Z Tang X Fu J Pan D Wang T Zhang Z. Case report: a complex multidisciplinary surgical approach for a pediatric patient with congenital meningoencephalocele. J Craniofac Surg. (2025) 36(6):e796–9. 10.1097/SCS.0000000000011534 40478249

[48] Kuiper BI Janssen LMJ Versteeg KS Ten Tusscher BL van der Spoel JI Lubbers WD Does preoperative multidisciplinary team assessment of high-risk patients improve the safety and outcomes of patients undergoing surgery? BMC Anesthesiol. (2024) 24(1):9. 10.1186/s12871-023-02394-5 38166642 PMC10759340

[49] Giugliano Villarroel C Ortiz Araya J Carvajal Guzmán M Ghazal Mohamad N. Nasoethmoidal dermoid cyst with intracranial extension. Case presentation and surgical management. J Craniofac Surg. (2025) 36(2):458–60. 10.1097/SCS.0000000000010853 39560951

[50] Crocetta FM Farneti P Sollini G Castellucci A Ghidini A Spinosi MC Endoscopic management of frontal sinus diseases after frontal craniotomy: a case series and review of the literature. Eur Arch Otorhinolaryngol. (2020) 278(4):1035–45. 10.1007/s00405-020-06335-7 32880737

[51] Purnell CA Skladman R Alden TD Corcoran JF Rastatter JC. Nasal dermoid cysts with intracranial extension: avoiding coronal incision through midline exposure and nasal bone osteotomy. J Neurosurg Pediatr. (2020) 25(3):298–304. 10.3171/2019.9.PEDS19132 31812133

[52] Morales Valencia E Tavares De La Paz LA Santos Vázquez G Serrano Padilla AE Moreno Pizarro E. Combined surgical approach to intracranial and extracranial hemangiopericytoma: case report and literature review. Cureus. (2020) 12(3):e7447. 10.7759/cureus.7447 32351826 PMC7186106

[53] Hsiao A Pimpalwar A. Subcutaneoscopic excision of external angular dermoid cysts: our covert scar approach in 11 cases. Eur J Pediatr Surg. (2018) 29(3):239–42. 10.1055/s-0038-1636942 29534256

[54] Adil E Rahbar R. Paediatric nasal dermoid: evaluation and management. Curr Opin Otolaryngol Head Neck Surg. (2021) 29(6):487–91. 10.1097/MOO.0000000000000765 34710067

[55] Amin SN Siu JM Purcell PL Manning JP Wright J Dahl JP Preoperative imaging and surgical findings in pediatric frontonasal dermoids. Laryngoscope. (2024) 134(4):1961–66. 10.1002/lary.31079 37776254

[56] Valencia-Sanchez BA Kim JD Zhou S Chen S Levy ML Roxbury C Special considerations in pediatric endoscopic skull base surgery. J Clin Med. (2024) 13:1924. 10.3390/jcm13071924 38610689 PMC11013018

[57] Bishop R Sheehan C Walz P Kern C Elmaraghy C. Management of infected nasal dermoid cysts and sinuses. J Surg Case Rep. (2021) 2021(4):rjab41. 10.1093/jscr/rjab041 PMC8024045 33854757

[58] Mazzola CA Null N. Multidisciplinary management of nasal dermoid with intracranial extension. J Clin Images Med Case Rep. (2023) 3:2766–7820. 10.52768/2766-7820/2080

[59] Donnell CC McKeague KF Cooper A Stenhouse J. Nasofrontal dermoid cyst: rare presentation of a philtrum sinus. Br J Oral Maxillofac Surg. (2020) 58(5):608–10. 10.1016/j.bjoms.2020.03.004 32201047

[60] Yılmaz Topçuoğlu M Plinkert PK Seitz A El Damaty A Bächli H Baumann I. A retrospective single-center study in 20 patients with midline nasal masses: which site has the highest risk of recurrence? Ann Otol Rhinol Laryngol. (2024) 134(3):218–24. 10.1177/00034894241300801 39610364 PMC11806645

[61] Riley CA Soneru CP Overdevest JB Otten ML Gudis DA. Pediatric sinonasal and skull base lesions. World J Otorhinolaryngol Head Neck Surg. (2020) 6(2):118–24. 10.1016/j.wjorl.2020.01.007 32596657 PMC7296510

[62] Karlsson J Lewis G Larsson P Lönnqvist P Diaz S. Intranasal dexmedetomidine sedation for paediatric MRI by radiology personnel: a retrospective observational study. Eur J Anaesthesiol. (2023) 40(3):208–15. 10.1097/EJA.0000000000001786 36546479

[63] Xie H Zhao J Tu H Wang W Hu Y. Combined sedation in pediatric magnetic resonance imaging: determination of median effective dose of intranasal dexmedetomidine combined with oral midazolam. BMC Anesthesiol. (2024) 24(1):112. 10.1186/s12871-024-02493-x 38521913 PMC10960491

[64] de Rover I Wylleman J Dogger JJ Bramer WM Hoeks SE de Graaff JC. Needle-free pharmacological sedation techniques in paediatric patients for imaging procedures: a systematic review and meta-analysis. Br J Anaesth. (2023) 130(1):51–73. 10.1016/j.bja.2022.09.007 36283870

[65] Mason KP Lubisch NB Robinson F Roskos R. Intramuscular dexmedetomidine sedation for pediatric MRI and CT. Am J Roentgenol. (2011) 197(3):720–25. 10.2214/AJR.10.6134 21862817

[66] Pham AH Le TD Chu HT Le TA Duong HD Van Dong H. Infected intradural dermoid cyst without dermal sinus tract mimicking brain abscess: a case report. Int J Surg Case Rep. (2020) 72:142–46. 10.1016/j.ijscr.2020.05.052 32535529 PMC7298525

[67] Lopez M Vermersch S Varlet F. Endoscopic excision of forehead and eyebrow benign tumors in children. J Laparoendosc Adv Surg Tech A. (2016) 26(3):226–30. 10.1089/lap.2015.0498 26870886

[68] Yao J Gong H Zhao X Peng Q Zhao H Yu S. Parental presence and intranasal dexmedetomidine for the prevention of anxiety during anesthesia induction in children undergoing tonsillectomy and/or adenoidectomy surgery: a randomized controlled trial. Front Pharmacol. (2022) 13:1015357. 10.3389/fphar.2022.1015357 36601054 PMC9806335

[69] Pierce CA Konofaos P Alvarez S Wallace RD. Excision and fat grafting of nasal tip dermoid cysts through an open rhinoplasty approach. J Craniofac Surg. (2016) 27(1):e18–20. 10.1097/scs.0000000000002291 26681174

[70] Anderson C Hamidian Jahromi A Miller EJ Konofaos P. The current status of the autologous fat grafting for pediatric craniofacial patients. Ann Plast Surg. (2020) 85(5):568–73. 10.1097/sap.0000000000002286 32049756

[71] Stier G Ramsingh D Raval R Shih G Halverson B Austin B Anesthesiologists as perioperative hospitalists and outcomes in patients undergoing major urologic surgery: a historical prospective, comparative effectiveness study. Perioper Med. (2018) 7:7–13. 10.1186/s13741-018-0090-y PMC6009851 29951203

[72] Liu H Li P Yu D Ma Z An Y Li S Analysis of the nursing effect of anesthesia care integration combined with preventive nursing on older patients with lumbar disc herniation during the perioperative period. Risk Manag Healthc Policy. (2023) 16:1001–09. 10.2147/rmhp.s411885 37323191 PMC10263014

[73] Soo KC Al Jajeh I Quah R Seah HKB Soon S Walker E. Virtual multidisciplinary review of a complex case using a digital clinical decision support tool to improve workflow efficiency. J Multidiscip Healthc. (2021) 20(14):1149–58. 10.2147/jmdh.s307470 PMC8147890 34045862

[74] Nadeem N Barrie J Bell R Shah N. P-P50 analysis of the efficiency of the hepatobiliary multidisciplinary team meeting to identify quality improvement strategies. Br J Surg. (2021) 108(Supplement_9):znab272–430. 10.1093/bjs/znab430.272

[75] de Carvalho INSR de Freitas RM Vargas FR. Translating micrornas into biomarkers: what is new for pediatric cancer? Med Oncol. (2016) 33(5):49. 10.1007/s12032-016-0766-4 27085875

[76] Liu Z Li C Xu J Lan Y Liu H Li X Crucial and overlapping roles of Six1 and Six2 in craniofacial development. J Dent Res. (2019) 98(5):572–79. 10.1177/0022034519835204 30905259 PMC6481007

[77] Xu J Liu H Lan Y Aronow BJ Kalinichenko VV Jiang R. A shh-foxf-fgf18-shh molecular circuit regulating palate development. Plos Genet. (2016) 12(1):e1005769. 10.1371/journal.pgen.1005769 26745863 PMC4712829

[78] Xu J Iyyanar PPR Lan Y Jiang R. Sonic hedgehog signaling in craniofacial development. Differentiation. (2023) 133:60–76. 10.1016/j.diff.2023.07.002 37481904 PMC10529669

[79] Ornitz DM Itoh N. New developments in the biology of fibroblast growth factors. Wires Mech Dis. (2022) 14(4):e1549. 10.1002/wsbm.1549 35142107 PMC10115509

[80] Tanay A Regev A. Scaling single-cell genomics from phenomenology to mechanism. Nature. (2017) 541(7637):331–38. 10.1038/nature21350 28102262 PMC5438464

[81] Khouri-Farah N Winchester EW Schilder BM Robinson K Curtis SW Skene NG Gene expression patterns of the developing human face at single cell resolution reveal cell type contributions to normal facial variation and disease risk. Biorxiv (2025):2021-25. 10.1101/2025.01.18.633396

[82] Aebersold R Mann M. Mass-spectrometric exploration of proteome structure and function. Nature. (2016) 537(7620):347–55. 10.1038/nature19949 27629641

[83] Doench JG Fusi N Sullender M Hegde M Vaimberg EW Donovan KF Optimized sgRNA design to maximize activity and minimize off-target effects of CRISPR-Cas9. Nat Biotechnol. (2016) 34(2):184–91. 10.1038/nbt.3437 26780180 PMC4744125

[84] Lambin P Leijenaar RTH Deist TM Peerlings J de Jong EEC van Timmeren J Radiomics: the bridge between medical imaging and personalized medicine. Nat Rev Clin Oncol. (2017) 14(12):749–62. 10.1038/nrclinonc.2017.141 28975929

[85] Jian Z Zhongjie S Yin K Bin W Xiyao L Hongwei Z. The application of augmented reality technology in endoscopic pituitary adenoma surgery via nasal approach. Gland Surg. (2025) 14(7):1318–35. 10.21037/gs-2025-95 40771379 PMC12322755

[86] Gul A Kucuker I Can E Niyaz L Yucel OE. A propranolol nonresponsive mass. J Craniofac Surg. (2015) 26(1):327–28. 10.1097/scs.0000000000001265 25502719

[87] Cho H Kim CK Park H. Overview of radiomics in prostate imaging and future directions. Br J Radiol. (2021) 95(1131):20210539. 10.1259/bjr.20210539 34797688 PMC8978251

[88] Liu L Cai W Zhou C Tian H Wu B Zhang J Ultrasound radiomics-based artificial intelligence model to assist in the differential diagnosis of ovarian endometrioma and ovarian dermoid cyst. Front Med. (2024) 11:1362588. 10.3389/fmed.2024.1362588 PMC10957533 38523908

[89] MacMahon P Iacob S Bach SE Elwood ET Lin JJ Avellino AM. Neurosurgical management of a rare congenital supratentorial neurenteric cyst with associated nasal dermal sinus: case report. J Neurosurg Pediatr. (2017) 20(6):521–25. 10.3171/2017.7.peds17123 28937919

